# The Effect of Magnesium Hydroxide Addition on the Extinguishing Efficiency of Sodium Bicarbonate Powders

**DOI:** 10.3390/ma15103449

**Published:** 2022-05-11

**Authors:** Piotr Izak, Mateusz Biel, Joanna Mastalska-Popławska, Paweł Janik, Piotr Mortka, Piotr Lesiak

**Affiliations:** 1 Faculty of Materials Science and Ceramics, AGH University of Science and Technology, Mickiewicza 30, 30-059 Krakow, Poland; izak@agh.edu.pl (P.I.); bielm@agh.edu.pl (M.B.); 2 Ogniochron S.A., Przemyslowa 42, 34-120 Andrychow, Poland; 3 Scientific and Research Centre for Fire Protection—National Research Institute, Nadwislanska 213, 05-420 Jozefow, Poland; pjanik@cnbop.pl (P.J.); pmortka@cnbop.pl (P.M.); plesiak@cnbop.pl (P.L.)

**Keywords:** BC extinguishing powder, magnesium hydroxide, extinguishing efficiency, thermal decomposition, powder rheology

## Abstract

This article analyzes the possibility of the modification of BC powder (a mixture of sodium bicarbonate and calcium carbonate) with magnesium hydroxide (Mg(OH)_2_). Extinguishing efficiency as well as the influence of this additive on other physicochemical properties were determined by performing a 13B fire test, rheological measurements of the powders, thermal tests (thermogravimetry (TG) and differential scanning calorimetry (DSC) in combination with quadrupole mass spectrometry (QMS)) and microscopic observations of the powders’ surface (scanning electron microscope (SEM) with energy dispersive X-ray analysis (EDS)). It was found that the increase of the Mg(OH)_2_ content causes deterioration of the rheological properties by increasing the slope angle of the flow curve in relation to the normal stress (the tangent of the flow curve slope varying from 0.258 for 5% of Mg(OH)_2_ up to 0.330 for 20% of Mg(OH)_2_). However, at the same time, the increased content of Mg(OH)_2_ increases the total energy of the chemical decomposition reaction (from −47.27 J/g for 5% of Mg(OH)_2_ up to −213.6 J/g for 20% of Mg(OH)_2_) resulting in the desirable higher level of heat removal from the fire. The initial extinguishing effect of the fire becomes more effective as the hydroxide content increases (within the first 2 s), but at a later stage (from t = 63 s), the temperature is no longer sufficient (it is below 350 °C) for thermal decomposition of Mg(OH)_2_. As such, the optimal content of Mg(OH)_2_ is 10–15%. The obtained results allowed for the assessment of the impact of individual powder components on its extinguishing effect and will contribute to the development of science in the field of developing new types of extinguishing powders.

## 1. Introduction

The powders available in portable firefighting equipment can, in principle, be divided into two groups: ABC powders suitable for extinguishing solids, flammable liquids and gases, and BC powders suitable for extinguishing flammable liquids and gases. Powder extinguishers are expected to be universal, affordable and easy to use, even by amateurs.

The extinguishing mechanism of the extinguishing powders depends on their chemical composition, microstructure and grain size. In the extinguishing process, a cloud of extinguishing powder is directed to the fire to lower the oxygen concentration, absorb heat and capture the radicals of chain reactions. According to modern theories, these radicals are the result of the pyrolytic decomposition of combustible materials, water or other reactions taking place in the fire environment which support the fire [1,2,3,4,5].

After the implementation of the Montreal Protocol (1987) [6], new solutions and new substances were searched for to be used in extinguishers instead of the previously commonly used halon. One of such compounds is magnesium hydroxide. Its addition can prevent powder agglomeration without unnecessary treatments and allow the extinguishing powders to penetrate a flame more effectively. Thanks to this, it can decrease the characteristic temperatures at different stages of the fire and absorb flame more quickly [7,8,9,10,11,12,13].

In the extinguishing process, apart from the mechanism of capturing the radicals of the chain reaction, the granulation of the powders, their particle size and surface modification also play a key role. Finer grinding/milling of the powders increases the total surface area, and thus creates more surface contact between free radicals present in the flame. Surface modification such as a coating can do likewise. As a result, the extinguishing powder allows for more effective neutralization of free radicals and inhibition of the combustion process [1,7,9,10,14,15,16,17,18,19,20,21].

Due to the above mechanism, the extinguishing efficiency depends on the number of products of the endothermic reactions created in the process of thermal decomposition in the gas phase and on the surface of the micrograins. Natural magnesium hydroxide is used commercially as a flame retardant. With a high degree of probability, this property can be attributed to the thermal decomposition of an endoenergetic nature at a temperature of approx. 330 °C. The heat absorbed during this reaction slows the burning process, and the released water hinders access to oxygen [1,8].

In this paper, components of the powder system are magnesium hydroxide, sodium bicarbonate and calcium carbonate. The last two compounds are commonly used in the extinguishing powders [22,23,24,25,26,27], but there is a lack of information about their mutual influence on the extinguishing effect. Hence, studies on the determination of the effect of magnesium hydroxide at the expense of sodium bicarbonate on both the fire extinguishing efficiency and the mechanism of action are justified. This article presents the possibility of replacing sodium bicarbonate with magnesium hydroxide and its results in terms of rheological and thermal properties, as well as the extinguishing efficiency of group B according to European standards. The obtained results will contribute to the development of the new types of extingusihing powders.

## 2. Materials and Methods

Four mixtures of BC type extinguishing powders consisting of magnesium hydroxide and sodium bicarbonate of various proportions, as well as calcium carbonate (constant amount) and an anti-caking agent in the amount of 5 wt%, were used for the tests. Powders were obtained from Caldic Deutschland GmbH, Am Karlshof 10, 40231 Dusseldorf. Their composition is shown in Table 1.

### Characterization of the BC Extinguishing Powders

**Figure 1:**
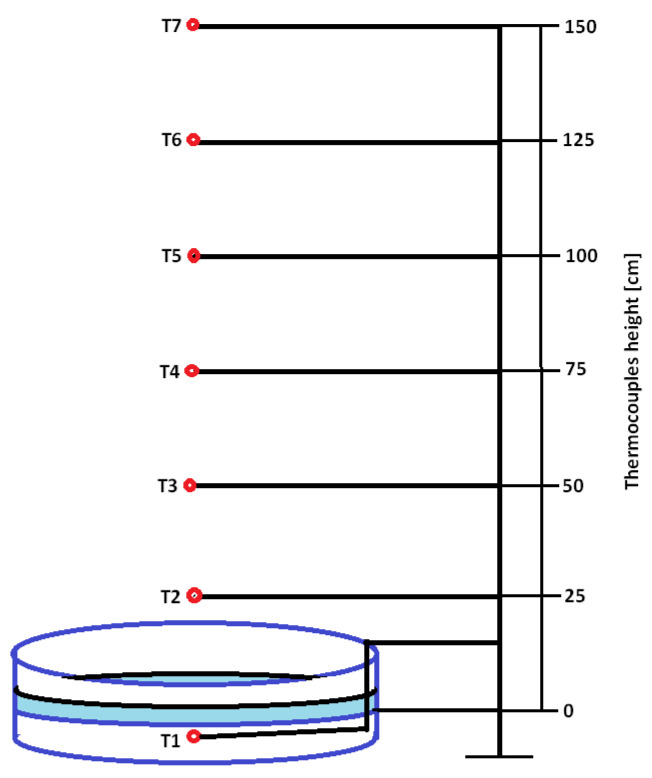
Scheme of the temperature measurement during the fire extinguishing test.

Extinguishing efficiency tests were carried out in accordance with the ISO7165 standard [28] by performing a 13B fire test. In total, 4 dm^3^ of water and 9 dm^3^ of heptane of parameters in accordance with the EN 3-7:2004 standard (distillation curve from 84 to 105 °C, the difference between the starting and ending point of distillation of ≤10 °C and the density at 15 °C in the range from 0.680 to 0.720 kg/dm^3^ [29]) were poured into a metal tray with a diameter of 740 ± 10 mm and a depth of 150 ± 5 mm. The fuel was set on fire and allowed to burn freely for 1 min. After that, extinguishing was started directing the stream of powder to the test fire. During the test, the time of extinguishing the fire and the amount of the remaining extinguishing powder were measured. In addition, the temperature above the flame was measured with the use of 7 thermocouples placed at heights of 0, 25, 50, 75, 100, 125 and 150 cm above the water layer (Figure 1).

The rheological measurements of the powder mixtures were performed with the use of a Brookfield Powder Flow Tester. The flow functions and the density curves as a function of normal stress, compared in terms of caking ability, were determined. The tests were carried out up to stress values of 8.6 kPa. The tested powders were intended for the so-called cartridge-operated fire extinguishers, into which1 kg of the powder was placed. The cylinder of these extinguishers had a diameter of 86 mm. The pressure exerted by 1 kg of the powder on the bottom of a fire extinguisher of this diameter was [29]:

(1) $$p=\frac{F}{S}=\frac{m\cdot g}{\frac{\pi \cdot d^{2}}{4}}=\frac{1\mathrm{kg}\cdot 9.81\frac{m}{s^{2}}}{\frac{\pi \cdot {\left(86\mathrm{mm}\right)}^{2}}{4}}=\frac{9.81N}{5808.8mm^{2}}=1.69\mathrm{kPa}$$

where *p* is the pressure exerted by the powder on the bottom of the fire extinguisher cylinder [kPa], *F* is the pressure force expressed as the product of the mass powder and gravity [N], and *S* is the pressure area expressed as the area of the round bottom of the fire extinguisher cylinder [mm^2^].

In view of the above, it was reasonable to conduct tests in the normal stress range of a few kPa. Due to the fact that the cartridge-operated fire extinguishers are pressurized devices only at the time of operation, the above calculations did not take into account the influence of the propellant gas. The self-compaction of the powder under its own weight had a much stronger influence on the flow of the powder.

Conducted thermal tests (thermogravimetry (TG) and differential scanning calorimetry (DSC) in combination with quadrupole mass spectrometry (QMS)) helped to compare the properties of the powder compositions in terms of the ability to remove heat from the fire, weight loss and gas identification. TG and DSC tests were carried out using a NETZSCH thermal analyzer, STA 449 F3 Jupiter model equipped with a QMS analyzer, heating the powder sample at a rate of 10 °C/min to a 1000 °C in corundum crucibles. During the test, the mass of the sample was measured, obtaining a thermogravimetric curve, and the determined DSC curve allowed for the identification of energy effects occurring during heating. In addition, the numberof released gases with specific atomic masses was measured using a QMS analyzer. The powders were also observed under the SEM NOVA NANO SEM 200 microscope cooperating with the EDS analyzer by EDAX. These tests were carried out in a low vacuum, and the powder samples were sputtered with carbon before microscopic observation.

## 3. Results and Discussion

### 3.1. Rheology of the Extinguishing Powders

From arheological point of view, powders can be divided into four groups: free-flowing, easy flowing, cohesive and very cohesive. The characteristic value qualifying the powder to a given group is the tangent of the flow curve slope to the normal stress axis. This value was calculated on the basis of the following equation [30]:

(2) $$tg\alpha =\frac{UFS_{\max}-UFS_{\min}}{MPCS_{\max}-MPCS_{\min}}$$

where *UFS*_max_ is the highest value of the unconfined failure strength [kPa], *UFS*_min_ is the lowest value of the unconfined failure strength [kPa], *MPCS*_max_ is the highest value of the major principal consolidating stress [kPa], and *MPCS*_min_ is the lowest value of the major principal consolidating stress [kPa].

The Jenike powder classification matrix is presented in Table 2 [30].

**Figure 2:**
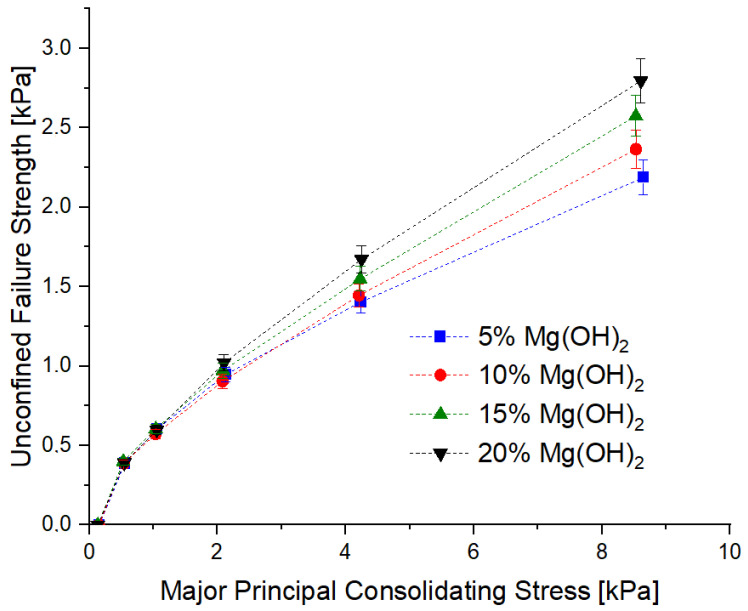
Flow curves of the tested powders.

The flow curves of the tested powders are shown in Figure 2.

**Figure 3:**
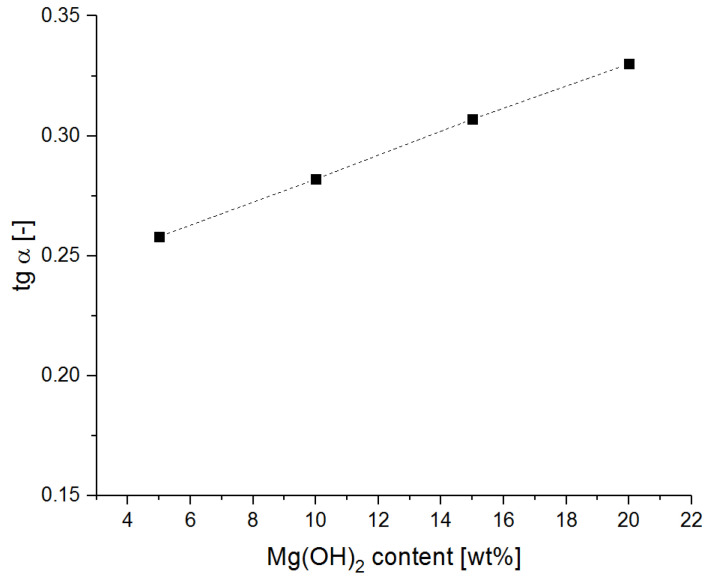
Value of the tgα of the flow curve as a function of the magnesium hydroxide content.

Values of the tgα calculated on the basis of Equation (2) and the classification of the powders in terms of their cohesiveness are shown in Table 3 and in Figure 3.

The conducted measurements show that all of the powders have a tendency to cake, i.e., self-adhere, especially at the consolidation pressure above 2 kPa. In the context of the application use of these powders, this is disadvantageous. This is because lower  values are expected from the extinguishing powders, which allows them to escape from the cylinder. However, in the self-compacting conditions in the cartridge-operated fire extinguishers, these differences are not significant. At the level of normal stress resulting from the pressure of the powder on the fire extinguisher bottom (approx. 1.69 kPa), all analyzed powder samples behaved similarly. As a result, these differences should not be apparent in the context of powder aeration.

**Figure 4:**
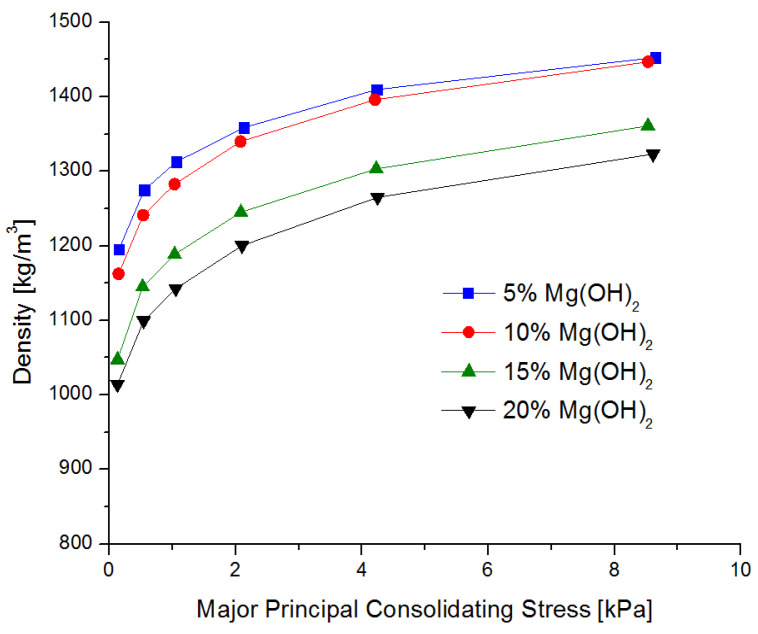
Dependence of density as a function of normal stress for the tested powder samples.

The compaction tendency of the powders was tested in a similar way. The compaction curves are shown in Figure 4.

**Figure 5:**
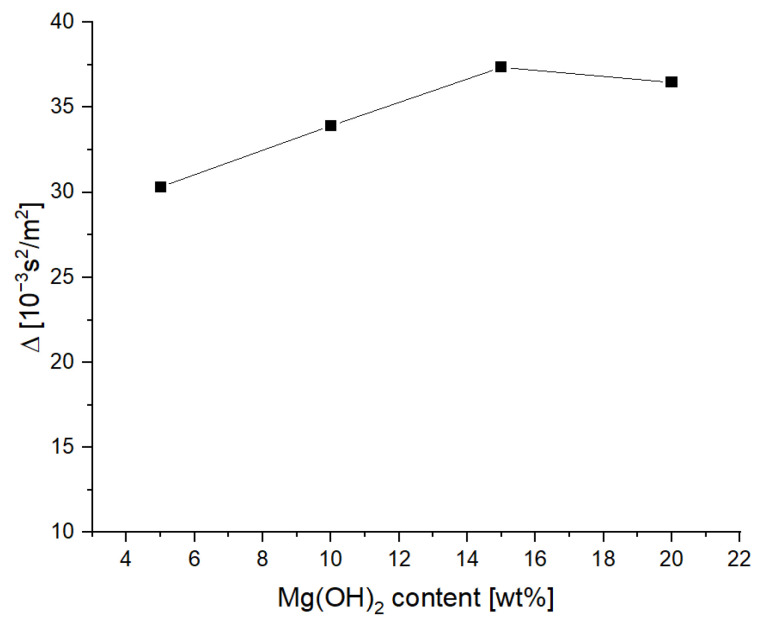
Kinetics of density changes as a function of normal stress of the tested powders.

The compaction kinetics, i.e., the Δ parameter, was determined according to Equation (3) [30]. The results of the compaction kinetics of the powders with different Mg(OH)_2_ content are presented in Table 4 and in Figure 5.

(3) $$\Delta =\frac{d_{\max}-d_{\min}}{MPCS_{\max}-MPCS_{\min}}[\frac{\mathrm{kg}}{m^{3}\cdot \mathrm{kPa}}=\frac{\mathrm{kg}\cdot m^{2}}{m^{3}\cdot 10^{3}N}=10^{-3}\frac{\mathrm{kg}}{m\cdot \mathrm{kg}\cdot \frac{m}{s^{2}}}=10^{-3}\frac{s^{2}}{m^{2}}]$$

where *d*_max_ is the highest measured density of the powder [kg/m^3^], *d*_min_ is the lowest measured density of the powder [kg/m^3^], *MPCS*_max_ is the highest value of the major principal consolidating stress [kPa], and *MPCS*_min_ is the lowest value of the major principal consolidating stress [kPa].

The value of the Δ parameter increases with the increase in the content of magnesium hydroxide, while after reaching a dozen or so mass percent, it stabilizes at the level of approx. 37 × 10^−3^ $\frac{s^{2}}{m^{2}}$ On the other hand, as the magnesium hydroxide content increases, the powder’s density decreases, leaving less space for the propellant gas. This can result in higher ejection energy of a powder containing a higher amount of magnesium hydroxide, which is particularly important in small fires of flammable liquids, where, in addition to the reaction with active radicals from the flame, blowing the flame out of the test fire tray is also expected.

### 3.2. TG and DSC Analysis

**Figure 6:**
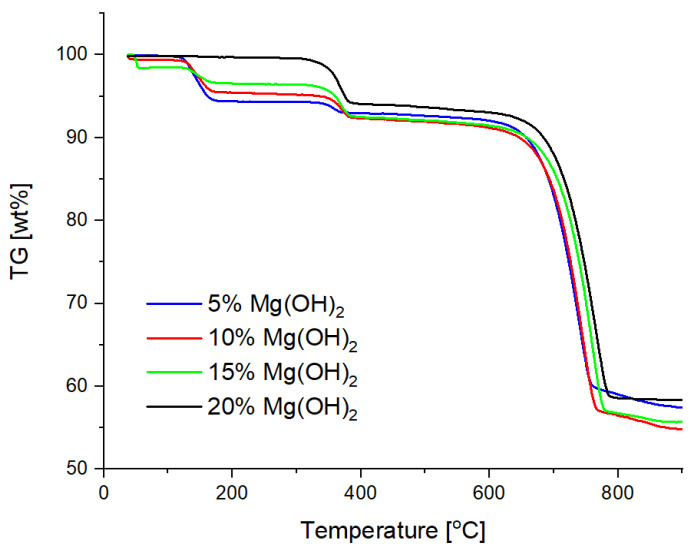
Comparison of the TG curves of the powder samples.

**Figure 7:**
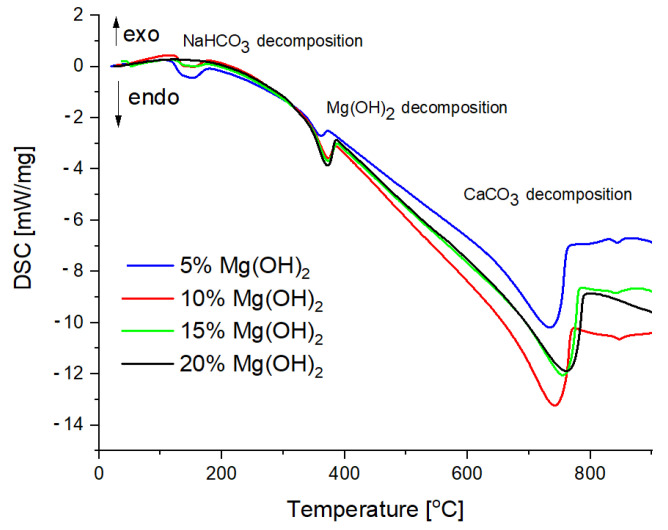
Comparison of the DSC curves of the powder samples.

TG curves are shown in Figure 6, whereas DSC curves are shown in Figure 7.

In the temperature range from 100 to 200 °C, the weight loss of the sample occurs due to the gases of 18 and 44 u detected by the QMS analyzer—these are probably gaseous products of sodium bicarbonate decomposition, i.e., carbon dioxide and water. Then, at a temperature of approx. 350 °C, there is a second weight loss of the sample with a gas release of 18 u. This effect is attributed to the thermal decomposition of magnesium hydroxide to magnesium oxide and water. The last effect is a large weight loss at approx. 700 °C, accompanied by a gas release of 44 u, which is attributed to the thermal decomposition of the calcium carbonate. While the decomposition effect of calcium carbonate is similar in individual samples, the decomposition effects of sodium bicarbonate and magnesium hydroxide are the more pronounced the more a given substance is in the sample [31].

**Figure 8:**
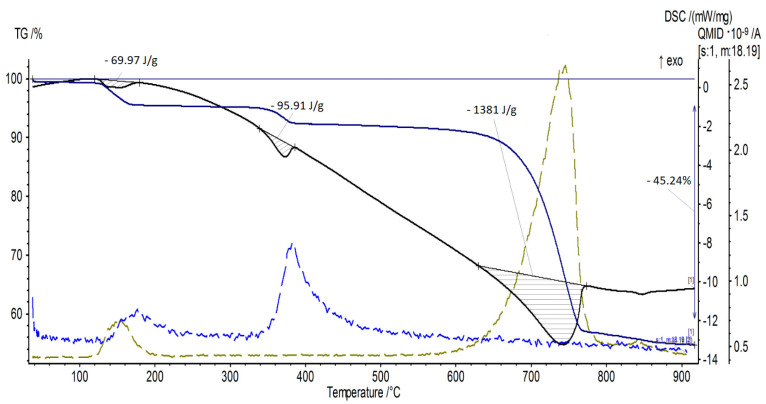
TG/DSC curve of the powder sample with 10 wt% of Mg(OH)_2_.

Figure 8 shows in detail both the presence and magnitude of individual energy effects as well as QMS curves showing the emission of gases with a mass of 18 and 44 u. The gas with a mass of 18 u was released in two temperature ranges: approx. 150–200 °C and approx. 350–400 °C. These ranges correspond to the thermal decomposition of sodium bicarbonate and magnesium hydroxide. This gas will be water. In turn, the gas with a mass of 44 u was released in the temperature ranges of approx. 150–200 °C and approx. 650–750 °C. The emission of this gas corresponds to the thermal decomposition of sodium bicarbonate and calcium carbonate. The gas will therefore be carbon dioxide [7,8,32].

Characteristic values of the energy effects from the TG/DSC measurements are presented in Table 5.

**Figure 9:**
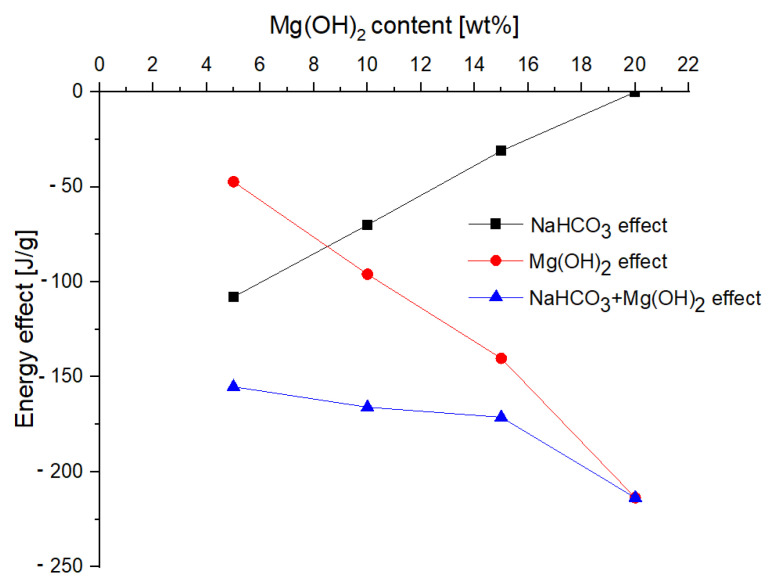
Comparison of the energetic effects of the decomposition of sodium bicarbonate and magnesium hydroxide.

As the content of magnesium hydroxide increases, the value of the endoenergetic effect of its decomposition also increases. Similarly, as a part of reducing the content of sodium bicarbonate, the value of its endoenergetic effect decreases. The comparison of these two effects, as well as their total energy, is presented in Figure 9.

When increasing the content of magnesium hydroxide to the level above 10%, a significant increase in the total energy effect of the thermal decomposition of the extinguishing powder mixture is observed. This effect is caused by the increasing share of the highly endoenergetic effect of the decomposition of magnesium hydroxide to the oxide and water. Based on the measurements, it can be concluded that the bigger the total energy effect is, the higher the share of magnesium hydroxide and the lower the sodium bicarbonate contents are. The energy effect of the decomposition of sodium bicarbonate is lower than that of magnesium hydroxide, therefore the extinguishing powder containing magnesium hydroxide may have better properties from the point of view of heat removal, considering the whole temperature range (0–900 °C) [9,10,11].

### 3.3. Scanning Electron Microscopy

**Figure 10:**
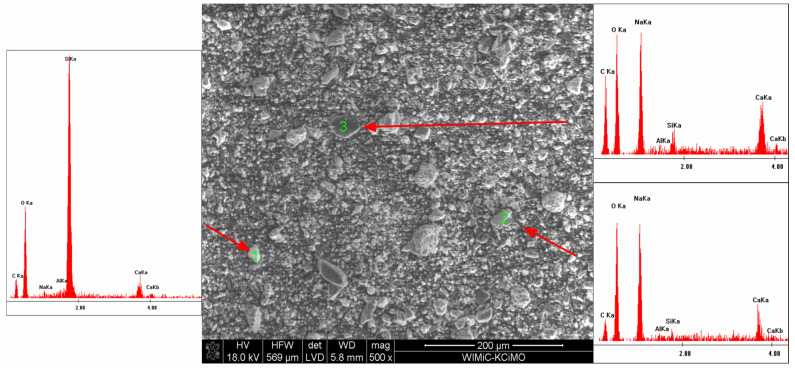
SEM microphotograph of the powder with 5 wt% of Mg(OH)_2_.

**Figure 11:**
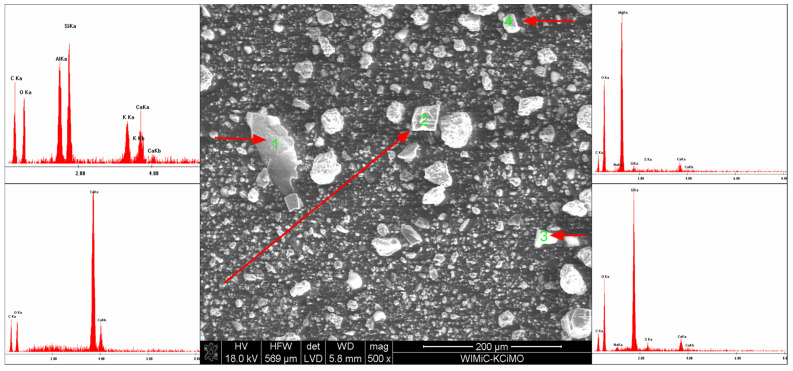
SEM microphotograph of the powder with 10 wt% of Mg(OH)_2_.

**Figure 12:**
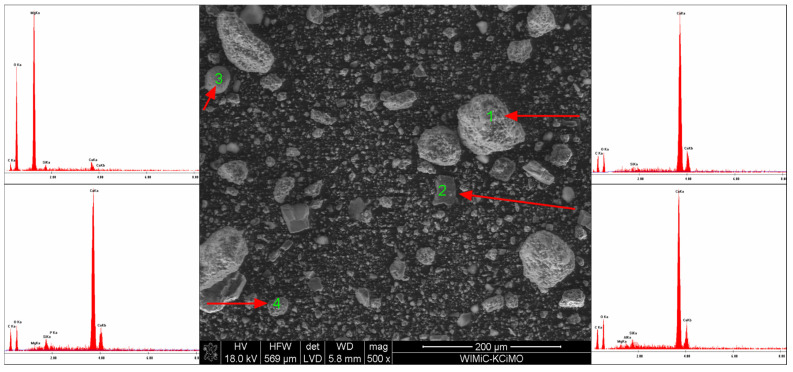
SEM microphotograph of the powder with 15 wt% of Mg(OH)_2_.

**Figure 13:**
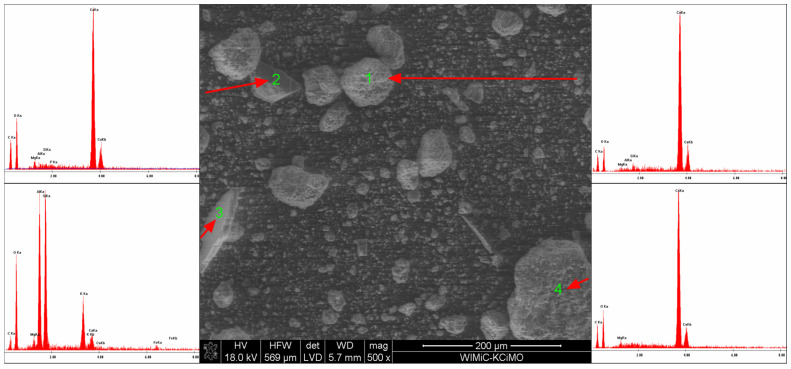
SEM microphotograph of the powder with 20 wt% of Mg(OH)_2_.

SEM microscope observations have shown that the powder grains have very diverse shapes, from curved to sharp-edged. EDS chemical analysis confirmed the existence of calcium carbonate, sodium bicarbonate and magnesium hydroxide, as well as sodium aluminosilicate. SEM microscopic photographs and the results of the EDS analysis are shown in Figure 10, Figure 11, Figure 12 and Figure 13.

### 3.4. Fire Tests

**Figure 14:**
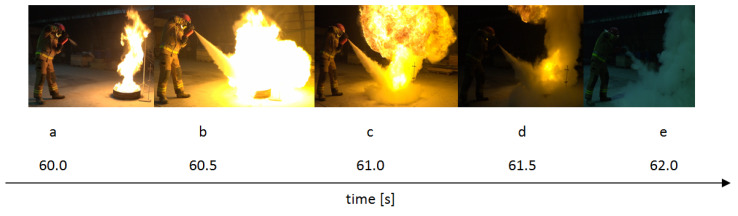
The course of the fire extinguishing with the use of a powder 1 sample: (**a**) 60 s, (**b**) 60.5 s, (**c**) 61 s, (**d**) 61.5 s, (**e**) 62 s after the flashover.

**Figure 15:**
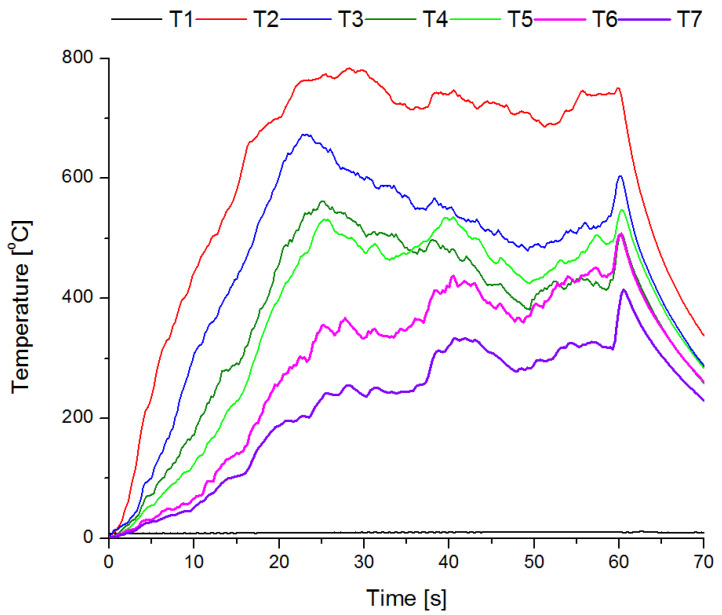
Fire temperature at various heights above the tray.

Fire tests were carried out in group B. Four fire tests were carried out for each of the powder mixtures. An exemplary course of fire extinguishing after 60 s from the flashover is shown in Figure 14, while an example of the temperature-time graph for a single measurement is shown in Figure 15. The temperatures were measured from the moment when the fire was ignited (t = 0 s) until the moment of extinction. A characteristic point is the maximum temperature 60 s after the flashover. At this point, the process of fire extinguishing with a one-kilogram fire extinguisher begins, the operating time of which was approx. 6–7 s. The temperature during the test was measured for approx. 70 s (60 s of fuel burning, 6–7 s of extinguishing, 3–4 s after extinguishing the fire).

Figure 15 shows that during the first 20 s of the test, the flashover occurs, and then in the range of 20–60 s the temperature stabilizes at each height of the thermocouples above the flame. After 60 s, the temperature drops quickly as a result of the extinguishing. After the flame disappears (maximum 67 s), the air temperature continues to decrease and after 70 s it is from approx. 230 °C to 340 °C, depending on the height above the tray. It can also be seen that the temperature of the water under the fuel layer does not change (9.5 ± 2 °C).

The obtained temperature measurement results were averaged, and then the kinetics of the temperature drop during the extinguishing process were calculated according to Equation (4) [30]:

(4) $$v_{i-j}=\frac{T_{i}-T_{j}}{t_{i}-t_{j}}[\frac{{}^{{}^{\circ}}C}{s}]$$

where *v*_*i*−*j*_ is the cooling rate [°C/s], *T_i_* is the thermocouple temperature in ‘*i*’ time (e.g., 60 s) [°C], *T_j_* is the temperature in ‘*j*’ time (e.g., 61 s) [°C], *t_i_* is the ‘*i*’ time (e.g., 60 s) [s], and *t_j_* is the ‘*j*’ time (e.g., 61 s) [s].

**Figure 16:**
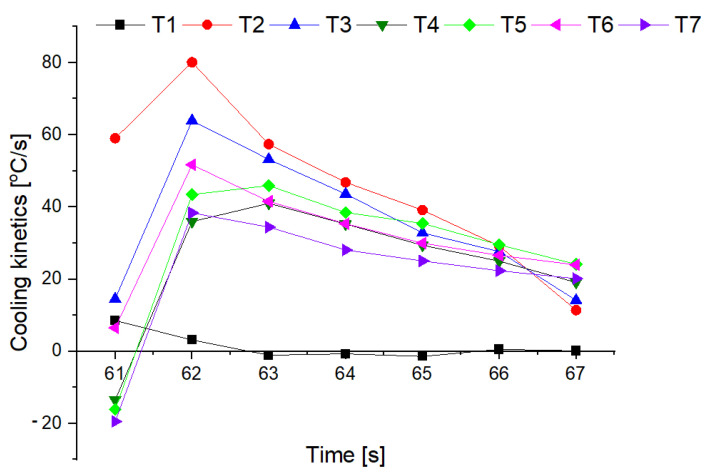
Cooling rate at an individual heights above the fire for a powder containing 5% of Mg(OH)_2_.

**Figure 17:**
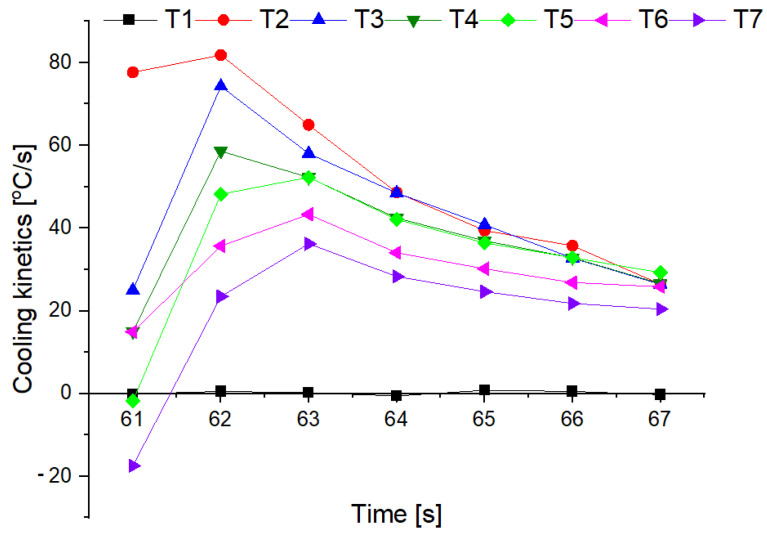
Cooling rate at an individual heights above the fire for a powder containing 20% of Mg(OH)_2_.

The changes in the cooling rate at different heights above the fire and for individual mixtures of the extinguishing powders are presented in Figure 16 and Figure 17.

The tests showed that the greatest cooling kinetics during the extinguishing of the test fire occurs within two seconds from the start of extinguishing, regardless of the height of the thermocouples above the tray and the type of powder. The extinguishing kinetics then decrease successively over time (Figure 16 and Figure 17). It can also be noticed that thermocouples at higher heights (above 75 cm) have a negative kinetic value, which means an increase in temperature at the initial extinguishing time. This is the result of the formation of a characteristic ‘fire mushroom’ (flashover) (Figure 14c). This is likely due to the addition (suction) of additional oxygen while the powder is being introduced to the fire. This phenomenon was observed in each extinguishing test. In the case of the powders containing 15% and 20% of magnesium hydroxide, negative values of the cooling rate at low heights were observed (25 cm and 50 cm above the fire). This proves that the fire temperature rises at the end of the extinguishing process, i.e., the fire has not been extinguished.

**Figure 18:**
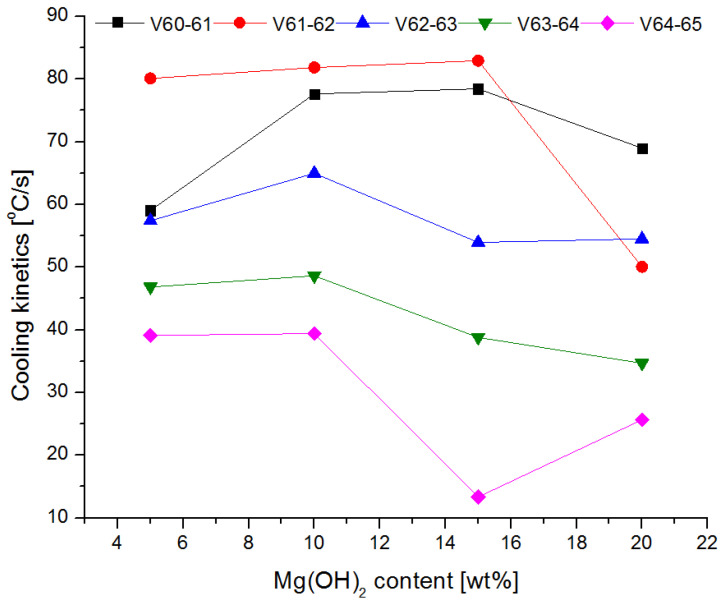
Cooling rate at 25 cm (thermocouple T2) above the fire depending on the Mg(OH)_2_content.

**Figure 19:**
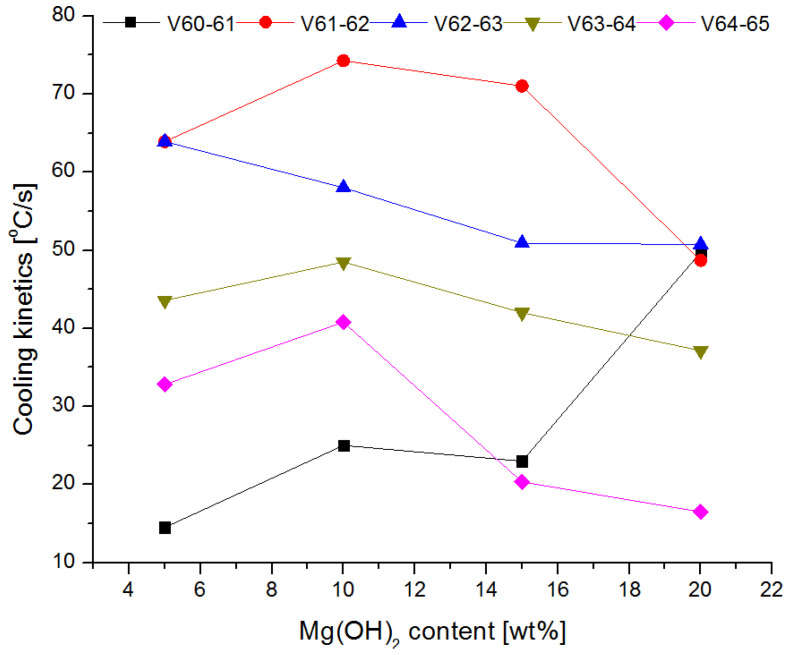
Cooling rate at 50 cm (thermocouple T3) above the fire depending on the Mg(OH)_2_content.

**Figure 20:**
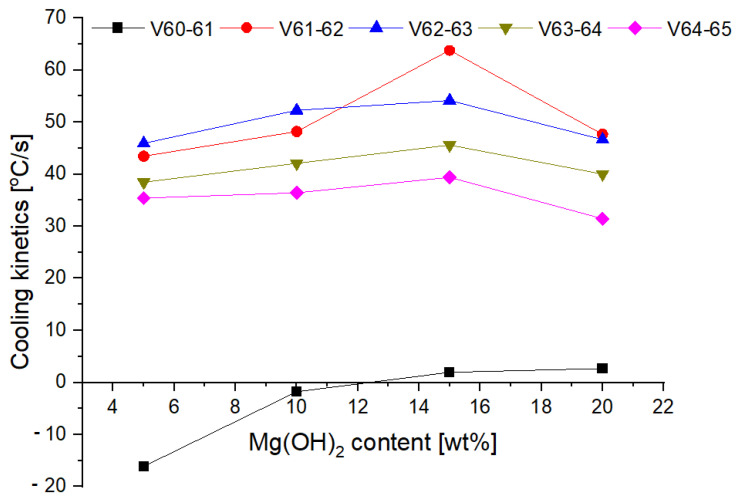
Cooling rate at 100 cm (thermocouple T3) above the fire depending on the Mg(OH)_2_content.

The calculated values of *v*_*i*−*j*_ for the thermocouples T2, T3 and T5 located 25, 50 and 100 cm from the substrate as a function of Mg(OH)_2_ content are presented in Figure 18, Figure 19 and Figure 20.

The cooling rate in the first second of extinguishing (v_60–61_) increases at each height above the fire, with a slight decrease at 25 cm above the fire for a powder containing 20% of Mg(OH)_2_. In further fire extinguishing phases (in particular from t = 63 s), the cooling rate is lower for powders containing a higher proportion of hydroxide. This behavior is due to fact that the fire temperature is too low at this extinguishing stage. The thermal decomposition of NaHCO_3_ occurs at a much lower temperature than the thermal decomposition of Mg(OH)_2_, the decomposition of which takes place at a temperature of approx. 350 °C. In the final stage of extinguishing the fire, the decreasing temperature prevents the thermal decomposition of Mg(OH)_2_, and consequently the heat reception and extinguishing of the fire. It is therefore a reasonable compromise to use a powder with a content of 10–15% of Mg(OH)_2_. This mixture allows for a much better heat reception at the beginning of fire extinguishing, and at the end of extinguishing, it shows similar properties. 

## 4. Conclusions

This work investigated the influence of magnesium hydroxide addition on the rheological and extinguishing properties of the BC extinguishing powders. On the basis of the analysis of the tests’ results, the following conclusions were made:

- The addition of magnesium hydroxide deteriorates the rheological properties and increases the ability to thicken and lump (the tangent of the flow curve slope varying from 0.258 for 5% of Mg(OH)_2_up to 0.330 for 20% of Mg(OH)_2_),which is disadvantageous from the point of view of using this compound as a fire extinguishing powder in popular portable fire extinguishing equipment;
- The addition of magnesium hydroxide significantly improves the properties of the powder from the point of view of heat reception (Mg(OH)_2_increases the total energy of the chemical decomposition reaction (from −47.27 J/g for 5% of Mg(OH)_2_ up to −213.6 J/g for 20% of Mg(OH)_2_)), taking into account the entire temperature range, which means that the use of this compound would allow faster heat transferfrom the fire, and thus extinguish the fire using smaller amounts of the extinguishing powder;
- The EDS analysis confirmed that the composition of the extinguishing powders, in addition to calcium carbonate, sodium bicarbonate and magnesium hydroxide, also includes sodium aluminosilicates and silica;
- The conducted test fires showed that the complete resignation of sodium bicarbonate in the composition of the extinguishing powder makes it difficult to extinguish the test fire in group B. When the fire temperature drops below 350 °C (from t = 63 s), magnesium hydroxide does not decompose and makes it difficult to collect the heat from the fire;
- The use of extinguishing powder containing 10–15% of magnesium hydroxide allows for better cooling and extinguishing properties, both at the beginning and at the end of the fire extinguishing process. In the first stage, the fire is extinguished by the decomposition of the magnesium hydroxide (when the temperature is above 350 °C), and finally by sodium bicarbonate (about 200 °C).

The conditions of the atomization of the extinguishing powder in the standard test of the fires from group B showed the formation of the flashover phenomenon, which inhibits its use in portable extinguishing equipment. It follows that the test fire should be extinguished while the extinguisher is in operation, i.e., 6–8 s, which is possible by the reduction of the temperature to 300 °C. It was obtained by the addition of the magnesium hydroxide. We are also conducting additional research to modify the fire tests, taking into account the phenomenon of powder extinguishing agent agglomeration, the flashover phenomenon, and the method of atomization.

## Figures and Tables

**Table 1:** Composition of the tested fire extinguishing powders.

Sample Number	Mg(OH)_2_ [wt%]	NaHCO_3_ [wt%]	CaCO_3_ [wt%]	Anti-Caking Agent [wt%]
1	5	15	75	5
2	10	10	75	5
3	15	5	75	5
4	20	0	75	5

**Table 2:** Classification of the powders from the flow function point of view.

Type of the Powder	tgα
Free flowing	<0.10
Easy flowing	<0.25
Cohesive	<0.50
Very cohesive	<1.00

**Table 3:** Classification of the tested powders.

Sample Symbol	tgα	Type of the Powder
5% Mg(OH)_2_	0.258	Cohesive
10% Mg(OH)_2_	0.282	Cohesive
15% Mg(OH)_2_	0.307	Cohesive
20% Mg(OH)_2_	0.330	Cohesive

**Table 4:** Δ values of the tested powder samples.

Mg(OH)_2_ Content [wt%]	Δ $\left[10^{-3}\frac{s^{2}}{m^{2}}\right]$
5	30.324
10	33.922
15	37.351
20	36.470

**Table 5:** Characteristic values of the individual effects from the TG/DSC measurements of the tested powder samples.

Sample Number	Mass Loss [wt%]	Decomposition of NaHCO_3_ [J/g]	Decomposition of Mg(OH)_2_ [J/g]	Decomposition of CaCO_3_ [J/g]	Total Energy Effect [J/g]
1	43.37	−107.9	−47.27	−1410	−1565.17
2	45.24	−69.97	−95.91	−1381	−1546.88
3	44.31	−30.99	−140.2	−1438	−1609.19
4	41.56	0	−213.6	−1582	−1795.60

## Data Availability

The data presented in this study are available within the article.

## References

[1] Hu W., Yu R., Chang Z., Tan Z., Liu X. (2021). The fire extinguishing mechanism of ultrafine composite dry powder agent containing Mg(OH)_2_. Int. J. Quantum Chem..

[2] Porshnov D., Ozols V., Ansone-Bertina L., Burlakovs J., Klavins M. (2018). Thermal decomposition study of major refuse derived fuel components. Energy Procedia.

[3] Yuen A.C.Y., Chen T.B.Y., Yeoh G.H., Yang W., Cheung S.C.-P., Cook M., Yu B., Chan Q.N., Yip H.L. (2018). Establishing pyrolysis kinetics for the modelling of the flammability and burning characteristics of solid combustible materials. J. Fire Sci..

[4] Nyazika T., Jimenez M., Samyn F., Bourbigot S. (2019). Pyrolysis modeling, sensitivity analysis, and optimization techniques for combustible materials: A review. J. Fire Sci..

[5] Chen T.B.Y., Cordeiro I.M.D.C., Yuen A.C.Y., Yang W., Chan Q.N., Zhang J., Cheung S.C.P., Yeoh G.H. (2022). An Investigation towards Coupling Molecular Dynamics with Computational Fluid Dynamics for Modelling Polymer Pyrolysis. Molecules.

[6] United Nations (2020). Montreal Protocol on Substances That Deplete the Ozone Layer.

[7] Liu H.-Q., Zong R.-W., Lo S., Hu Y., Zhi Y.-R. (2018). Fire Extinguishing Efficiency of Magnesium Hydroxide Powders under Different Particle Size. Procedia Eng..

[8] Du D., Shen X., Feng L., Hua M., Pan X. (2018). Efficiency characterization of fire extinguishing compound superfine powder containing Mg(OH)_2_. J. Loss Prev. Process Ind..

[9] Du D.-X., Pan X.-H., Hua M. (2018). Experimental Study on Fire Extinguishing Properties of Compound Superfine Powder. Procedia Eng..

[10] Ibrahim H., Patruni J.R. (2020). Experimental investigation on extinguishing performance of a novel nanocomposite for gaseous fires. J. Loss Prev. Process Ind..

[11] Wang Z., Meng X., Yan K., Ma X., Xiao Q., Wang J., Bai J. (2020). Inhibition effects of Al(OH)_3_ and Mg(OH)_2_ on Al-Mg alloy dust explosion. J. Loss Prev. Process Ind..

[12] Huang C., Chen X., Yuan B., Zhang H., Shang S., Zhao Q., Dai H., He S., Zhang Y., Niu Y. (2020). Insight into suppression performance and mechanisms of ultrafine powders on wood dust deflagration under equivalent concentration. J. Hazard. Mater..

[13] Huang C., Chen X., Yuan B., Zhang H., Dai H., He S., Zhang Y., Niu Y., Shen S. (2019). Suppression of wood dust explosion by ultrafine magnesium hydroxide. J. Hazard. Mater..

[14] Fan R., Jiang Y., Li W., Xiong C., Qiu R. (2019). Investigation of the physical and chemical effects of fire suppression powder NaHCO_3_ addition on methane-air flames. Fuel.

[15] Luo Z., Su Y., Chen X., Zheng L. (2019). Effect of BC powder on hydrogen/methane/air premixed gas deflagration. Fuel.

[16] Chen X., Zhang H., Chen X., Liu X., Niu Y., Zhang Y., Yuan B. (2017). Effect of dust explosion suppression by sodium bicarbonate with different granulometric distribution. J. Loss Prev. Process Ind..

[17] Ibrahim H., Patruni J.R. (2020). Experimental assessment on LPG fire extinguishing properties of three chemical powders before and after milling action. Fire Mater..

[18] Huang D., Wang X., Yang J. (2015). Influence of Particle Size and Heating Rate on Decomposition of BC Dry Chemical Fire Extinguishing Powders. Part. Sci. Technol..

[19] Boroujerdnia M., Obeydavi A., Sabzi M. (2021). Synthesis and characterisation of a novel Fe-based nanocomposite by mechanical alloying and spark plasma sintering. Powder Met..

[20] Bahrami A., Mohammadnejad A., Sajadi M. (2021). Microstructure and mechanical properties of spark plasma sintered AlCoFeMnNi high entropy alloy (HEA)-carbon nanotube (CNT) nanocomposite. J. Alloys Compd..

[21] Sabzi M., Anijdan S.M., Ghobeiti-Hasab M., Fatemi-Mehr M. (2018). Sintering variables optimization, microstructural evolution and physical properties enhancement of nano-WC ceramics. J. Alloys Compd..

[22] Yan K., Meng X. (2020). An investigation on the aluminum dustexplosion suppression efficiency and mechanism of a NaHCO_3_/DE composite powder. Adv. Powder Technol..

[23] Miao N., Zhong S., Yu Q. (2015). Ignition characteristics of metal dusts generated during machining operations in the presence of calcium carbonate. J. Loss Prev. Process Ind..

[24] Zhao J., Xue F., Fu Y., Cheng Y., Yang H., Lu S. (2021). A comparative study on the thermal runaway inhibition of 18650 lithium-ion batteries by different fire extinguishing agents. iScience.

[25] Birchall J. (1970). On the mechanism of flame inhibition by alkali metal salts. Combust. Flame.

[26] Jensen D.E., Jones G.A. (1982). Kinetics of flame inhibition by sodium. J. Chem. Soc. Faraday Trans..

[27] Jiang H., Bi M., Peng Q., Gao W. (2019). Suppression of pulverized biomass dust explosion by NaHCO_3_ and NH_4_H_2_PO_4_. Renew. Energy.

[28] (2017). Fire Fighting-Portable Fire Extinguishers-Performance and Construction.

[29] (2007). Portable Fire Extinguishers—Part 7: Characteristics, Performance Requirements and Test Methods.

[30] Yan Q.-L., He G.-Q., Liu P.-J., Gozin M. (2019). Nanomaterials in Rocket Propulsion Systems.

[31] Schroeder S.L.M., Gottfried M. Temperature-Programmed Desorption (TPD) Thermal Desorption Spectroscopy (TDS). http://www.uhv.es/sites/marte/includes/doc/tds.pdf.

[32] Koshiba Y., Haga T., Ohtani H. (2019). Flame inhibition by calcium compounds: Effects of calcium compounds on downward flame spread over solid cellulosic fuel. Fire Saf. J..

